# The adverse impact of herbicide Roundup Ultra Plus in human spermatozoa plasma membrane is caused by its surfactant

**DOI:** 10.1038/s41598-022-17023-3

**Published:** 2022-07-29

**Authors:** Mercedes Torres-Badia, Soraya Solar-Malaga, Rebeca Serrano, Luis J. Garcia-Marin, M. Julia Bragado

**Affiliations:** Research Group of Intracellular Signaling and Technology of Reproduction (SINTREP), Department of Biochemistry and Molecular Biology and Genetics, Instituto de Investigación INBIO G+C, Universidad de Extremadura, 10003 Cáceres, Spain

**Keywords:** Reproductive biology, Urology

## Abstract

The scarce research about the worldwide used glyphosate-based herbicide Roundup is controversial in human reproduction, especially spermatozoa. This study investigates the in vitro effect in human spermatozoa of Roundup Ultra Plus (RUP), its active ingredient glyphosate and its non-active, surfactant. Human spermatozoa were incubated (1 h, 37 °C) in presence/absence of RUP 0.01%, glyphosate, or equivalent surfactant concentration. Motility and sperm parameters were analyzed by C.A.S.A and flow cytometry, respectively. RUP significantly increases sperm plasma membrane lipid disorganization in a concentration-dependent manner while it decreases plasma membrane integrity. RUP significantly increases the death spermatozoa population after A23187-induced acrosome reaction, without affecting sperm viability, mitochondrial membrane potential, ROS content, acrosome membrane damage, phosphatidylserine exposure, A23187-induced acrosome reaction or GSK3 phosphorylation. RUP also significantly decreases motile and the a + b sperm populations. Interestingly, all sperm effects caused by RUP 0.01% are mimicked by its surfactant POEA at equivalent concentration. However, glyphosate does not affect any sperm parameter, even using 10-times higher concentration than the RUP 0.01% equivalent. RUP disturbs lipid organization and integrity of human sperm plasma membrane and reduces motility, without affecting viability or functional parameters. Importantly, RUP adverse effects in human spermatozoa are caused by the surfactant and no by glyphosate.

## Introduction

The active ingredient of the broad-spectrum Roundup herbicide is glyphosate, *N*-(phosphonomethyl) glycin, which inhibits the enzyme 5-enolpyruvylshikimate-3-phosphate synthase in plants leading to a blockade in the synthesis of essential amino acids^1^. This commercial herbicide is not only among the most used worldwide in agriculture, but also its massive use is growing every year about 20%. This represents a serious risk because glyphosate-based herbicides severely contaminate the environment (water, soil, and ecosystems) and therefore a wide concern for animal and human health has emerged based on the consequences of its vast exploitation. Beside the active compound, other non-active ingredients are included in the commercial Roundup formulation, such as surfactant molecules. Initially, potential noxious effects of glyphosate-based herbicides in mammalian species^2–4^ have been mostly attributed to glyphosate, which at low doses might act as an endocrine disruptor, leading to an impairment of hormones physiological function^3,5^. However, it has been also demonstrated in different human cells^6–9^, including embryonic^10,11^ and placental cells^10–12^, that the harmful effects of Roundup are higher than the effects of glyphosate alone, suggesting that besides glyphosate, other Roundup components must be involved in the adverse impact of the herbicide in mammalian cells.

In male reproductive cells, the effect of Roundup has been studied in animal^2,13,14^ and human species^15,16^, and results about its toxicity are controversial. Thus, Cassault-Meyer et al.^13^ found that glyphosate exposure in rats did not affect sperm concentration, viability or motility, whereas a meta-analysis in rodents showed that glyphosate resulted harmful and reduced sperm concentration^2^. A recent in vitro study in stallion spermatozoa reported that Roundup is more toxic than its active molecule glyphosate, which did not induce any detrimental impact on sperm quality^17^. Moreover, this study pointed the Roundup detrimental effects mainly at membrane and mitochondria. Another in vitro study comparing Roundup and its active component in pig spermatozoa reports that while both Roundup and glyphosate have adverse effects on sperm function and survival, Roundup is more toxic than glyphosate^4^. In fact, low Roundup concentrations similar to those present as environment contaminants, impair pig sperm motility likely due to a detrimental effect in plasma membrane lipid organization and to inhibition of phosphorylation of both, GSK3β and specific PKA substrates^14^. Interestingly, this recent finding clearly shows that adverse effects of low RUP concentrations in pig spermatozoa function are caused by the surfactant POEA included in its commercial formulation and not at all by its active ingredient glyphosate^14^.

In human reproductive tissues, there are also controversial data. Whereas Williams et al.^18^ reported no evidence about an adverse effect of glyphosate, in vitro exposure of human spermatozoa to 1 μg mL^−1^ Roundup reduces sperm motility, likely through mitochondrial deregulation^16^. Unlike in the pig using different concentrations^14^, these Roundup adverse effects in human spermatozoa seem to be mimicked by the equivalent glyphosate concentration (0.36 μg mL^−1^)^16^. In fact, it has been recently reported that glyphosate at 0,1 μM and 1 μM negatively affects the mitochondrial respiratory control ratio by decreasing the rates of oxygen consumption in the active and resting states of mitochondrial respiration in human spermatozoa^19^. Furthermore, this effect was increased in the presence of di-hydroxytestosterone, although the mechanism of glyphosate action in mitochondria is unknown^19^. More research on the consequences of the exposure of Roundup herbicide and its individual ingredients in human male gamete is needed, as the specific effect of the surfactants included in its commercial formulation, has not been addressed experimentally together with Roundup and glyphosate in human spermatozoa. Therefore, the objective of this work was to investigate the functional impact of in vitro exposure of human spermatozoa to the herbicide Roundup, to its active ingredient glyphosate and to a non-active ingredient, a surfactant (the principal adjuvant according to the fabricator). This work used low Roundup Ultra Plus concentrations 100 times lower than advised for agricultural employ, similar to those present as environment contaminants, which allowed us to study its possible adverse impact, as well as its active and non-active ingredients alone or combined, in human sperm function.

## Results

### Effects of Roundup Ultra Plus (RUP), its active component, potassium glyphosate (GLY) and surfactant (polyoxyethylene amine, POEA), on human sperm viability

Initially, we studied the viability of human spermatozoa exposed (1 h, 5% CO_2_, 37 °C) to RUP (0.01%), corresponding to the highest concentration used in our previous work investigating the herbicide Roundup Ultra Plus action on other mammalian spermatozoa, such as pig^14^.

**Figure 1:**
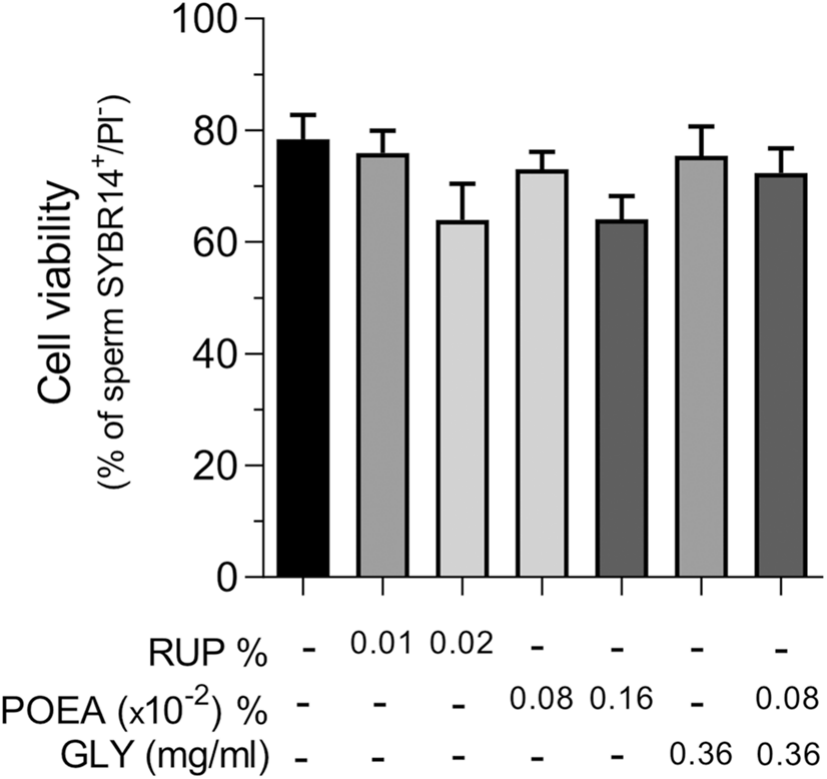
Effects of Roundup Ultra Plus (RUP), surfactant polyoxyethylene tallow amine (POEA) and Glyphosate (GLY) in human sperm viability. Spermatozoa were incubated in BWW for 1 h at 37 °C, 5% CO_2_, in the absence or presence of different concentrations of RUP (0.01%, 0.02%), POEA (0.0008%, 0.0016%), GLY (0.36 mg mL^−1^) or POEA (0.0008%) in combination with GLY (0.36 mg mL^−1^). This experiment was performed 6 times (n = 6) and the results were expressed as the mean of the percentage of SYBR14-positive and PI-negative spermatozoa ± standard error of the mean (SEM). No statistical differences are found.

As shown in Fig. 1, this concentration did not alter cell viability, nor did the double RUP concentration (0.02%), although a dose-dependent trend was perceived. When RUP concentrations were 10 times higher (0.1%) total sperm mortality was found (data not shown). Simultaneously, we investigated the viability of spermatozoa treated with GLY concentration of 0.36 mg mL^−1^, which is equivalent to the highest RUP concentration (0.1%) and no significant differences were found (Fig. 1). Then, we studied the effects of other RUP component, the surfactant POEA, under the same experimental conditions. The sperm treatment with POEA 0.008%, equivalent to RUP 0.1%, caused total mortality of spermatozoa (data not shown). In contrast, the viability of sperm samples exposed to concentrations of POEA 0.0008% and 0.0016%, which are equivalent to RUP 0.01% and 0.02%, respectively, were not statistically affected, although a dose-dependent trend was perceived (Fig. 1). Finally, we investigated the effect of the combination of both RUP ingredients, GLY (0.36 mg mL^−1^) and POEA (0.0008%), and as seen in Fig. 1, this combined treatment did not statistically affect sperm viability.

### Effects of Roundup Ultra Plus (RUP) and its components, GLY and surfactant POEA, on plasma membrane fluidity of human spermatozoa

**Figure 2:**
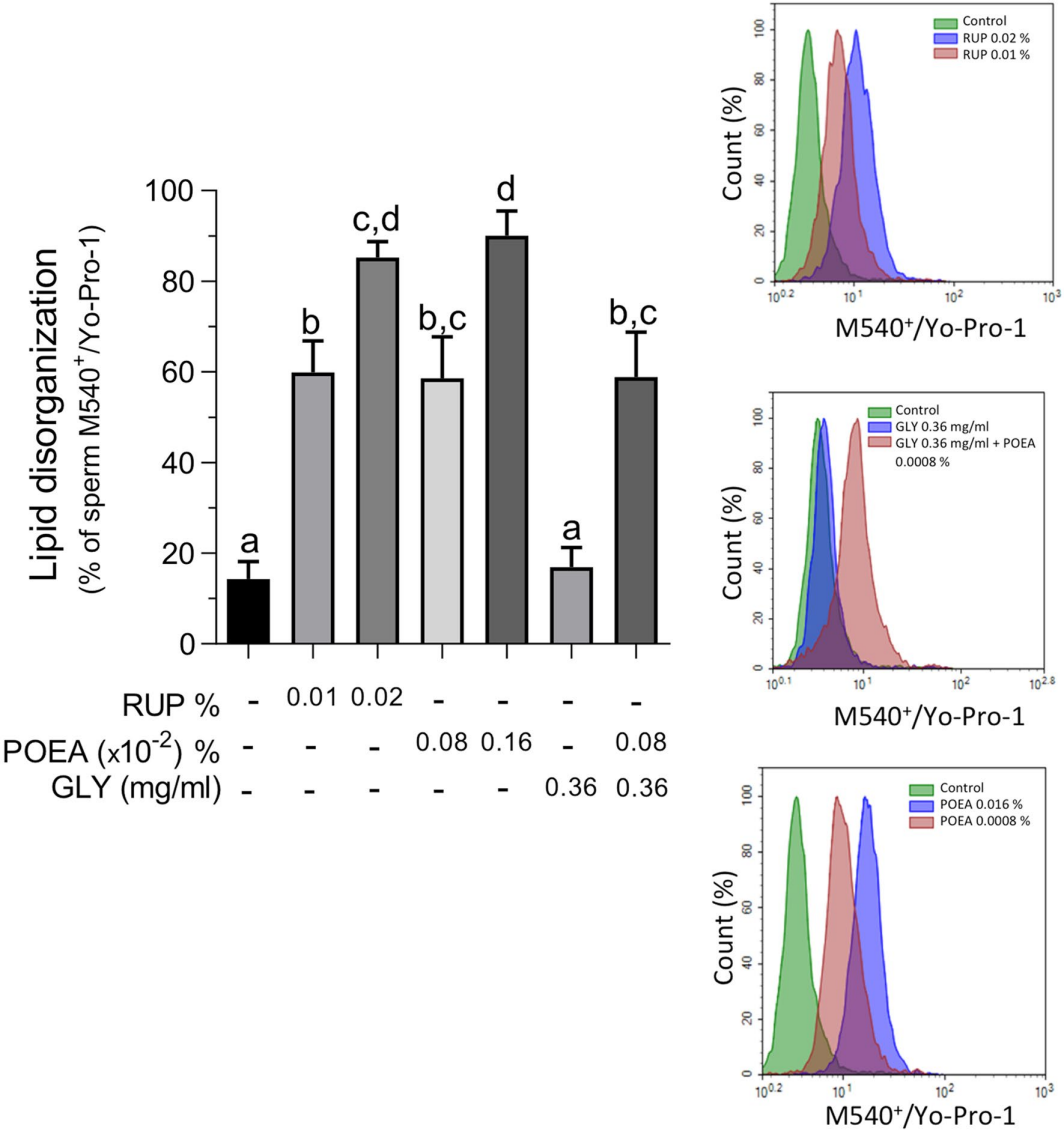
Effects of Roundup Ultra Plus (RUP), surfactant polyoxyethylene tallow amine (POEA) and Glyphosate (GLY) in plasma membrane lipid organization of human spermatozoa. Spermatozoa were incubated in BWW for 1 h at 37 °C, 5% CO_2_, in the absence or presence of indicated concentrations of RUP, POEA or GLY. Left panel: results were expressed as the mean of the percentage of M540-positive and Yo-Pro-1-negative. Right panel: flow cytometry histograms plots showing fluorescence of M540-positive and Yo-Pro-1-negative spermatozoa of sperm samples incubated in absence or presence of the indicated concentrations of RUP (upper panel), GLY or POEA (middle and lower panels). Each experiment was performed at least 12 times (n = 12). Columns with different letters are statistically different from each other, so that for P < 0.05.

Previously, it has been demonstrated that the main effect of RUP in other mammalian spermatozoa, as pig^14^ and stallion^17^ was at the plasma membrane. Therefore, we evaluated the effect of RUP on the plasma membrane lipid organization of human spermatozoa at those concentrations that unaffected sperm viability. Human spermatozoa incubated (1 h, 5% CO_2_, 37 °C) with RUP 0.01% and 0.02%, showed a clear and statistically significant increase in the plasma membrane lipid disorganization in a dose-dependent manner (Fig. 2). Similarly, when spermatozoa were exposed to the surfactant POEA at equivalent concentrations of RUP, a statistically significant increase was detected in plasma membrane disorganization in a dose-dependent way (Fig. 2). However, incubation of human spermatozoa with 0.36 mg mL^−1^ GLY, which is equivalent to the highest RUP dilution (0.1%), showed no differences from control. Interestingly, the combination of GLY 0.36 mg mL^−1^ and POEA 0.0008% did not potentiate the POEA effect alone on membrane lipid disorganization (Fig. 2).

**Figure 3:**
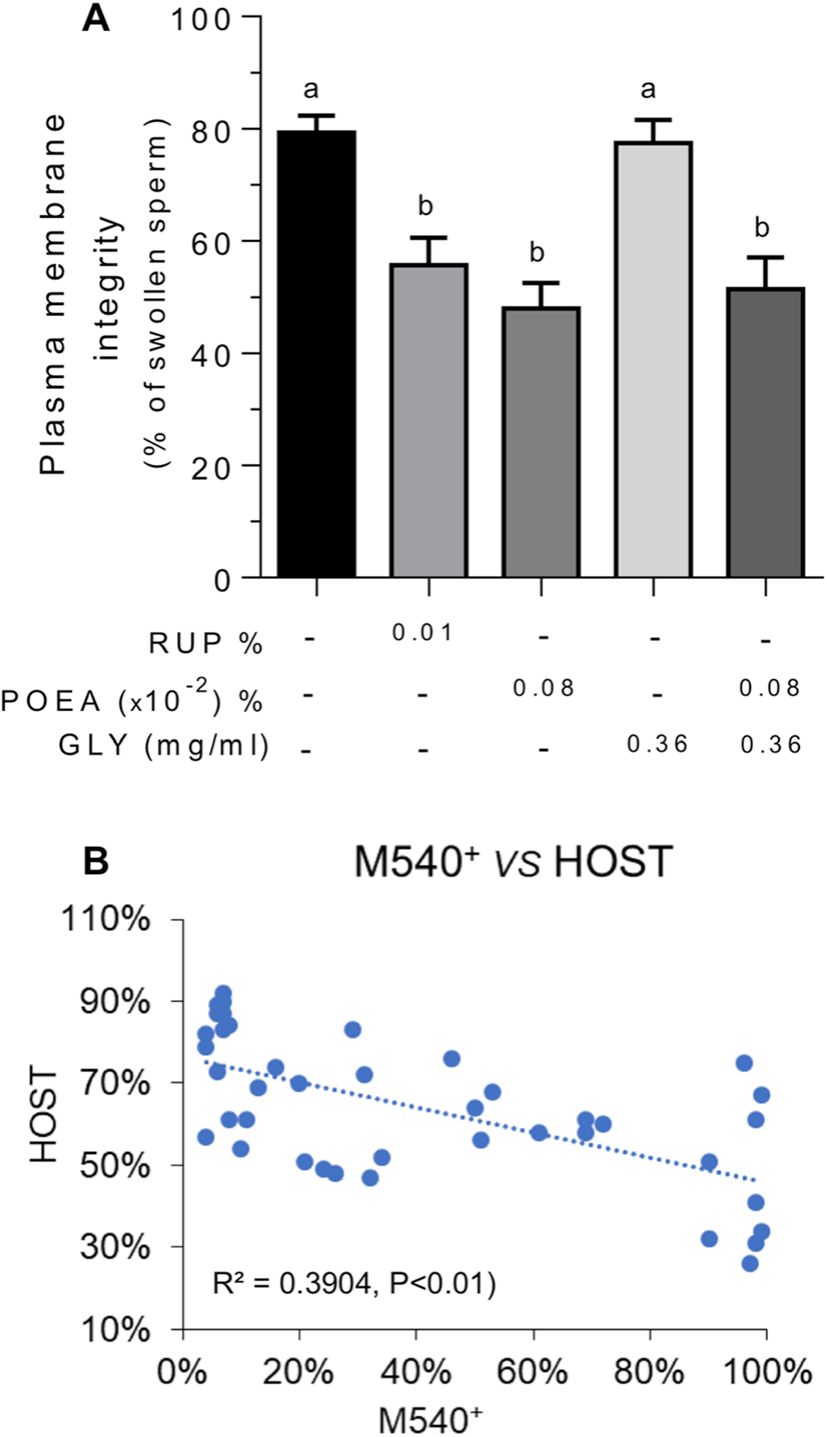
Effects of Roundup Ultra Plus (RUP), glyphosate (GLY) and surfactant polyoxyethylene tallow amine (POEA) in functional integrity of human sperm plasma membrane (HOS test). (**A**) Spermatozoa were incubated in BWW for 1 h at 37 °C, 5% CO_2_, in the absence or presence of indicated concentrations of RUP, POEA, GLY or POEA in combination with GLY. This experiment was performed 7 times (n = 7) and the results were expressed as the mean of the percentage of swollen spermatozoa ± standard error of the mean (SEM). Columns with different letters are statistically different from each other, so that for P < 0.05. (**B**) Negative correlation between the lipid disorganization (M540-positive and Yo-Pro-1-negative spermatozoa) and the functional integrity (HOS test) of the human sperm plasma membrane. Values were result of 7 independent experiments (n = 35). The resultant Pearson coefficient is statistically significant for the negative correlation, (r = − 0.625, *P* < 0.05).

Next, we investigated the functional integrity of the human sperm membrane through the HOS test by screening the sperm membrane’s ability to maintain balance in a hypo-osmotic medium^20^. Thus, a higher percentage of swollen spermatozoa indicates the presence of spermatozoa having a functional and intact plasma membrane. Incubation of human spermatozoa with RUP 0.01% and POEA 0.0008% caused a significant decrease in the percentage of swollen spermatozoa. A statistically significant decrease was also observed after POEA 0.0008% incubation (Fig. 3A). However, incubation with GLY 0.36 mg mL^−1^ showed no differences respect to the control. Finally, the combined treatment of GLY 0.36 mg mL^−1^ and POEA 0.0008% did not decrease the percentage of rolled tails spermatozoa, which was similar to the obtained after POEA treatment alone (Fig. 3A).

Our results showed that the higher percentage of sperm M540^+^/Yo-Pro-1^−^, the lower was the percentage of swelling and curling of the tail of spermatozoa under hypo-osmotic conditions. Therefore, we next established in Fig. 3B a statistically significant negative correlation between plasma membrane lipid disorganization, evaluated through flow cytometry, with the double stain with M540 and Yo-Pro-1, and the functional response of the sperm membranes, studied through the HOS test (r = − 0.625, *P* < 0.05).

**Figure 4:**
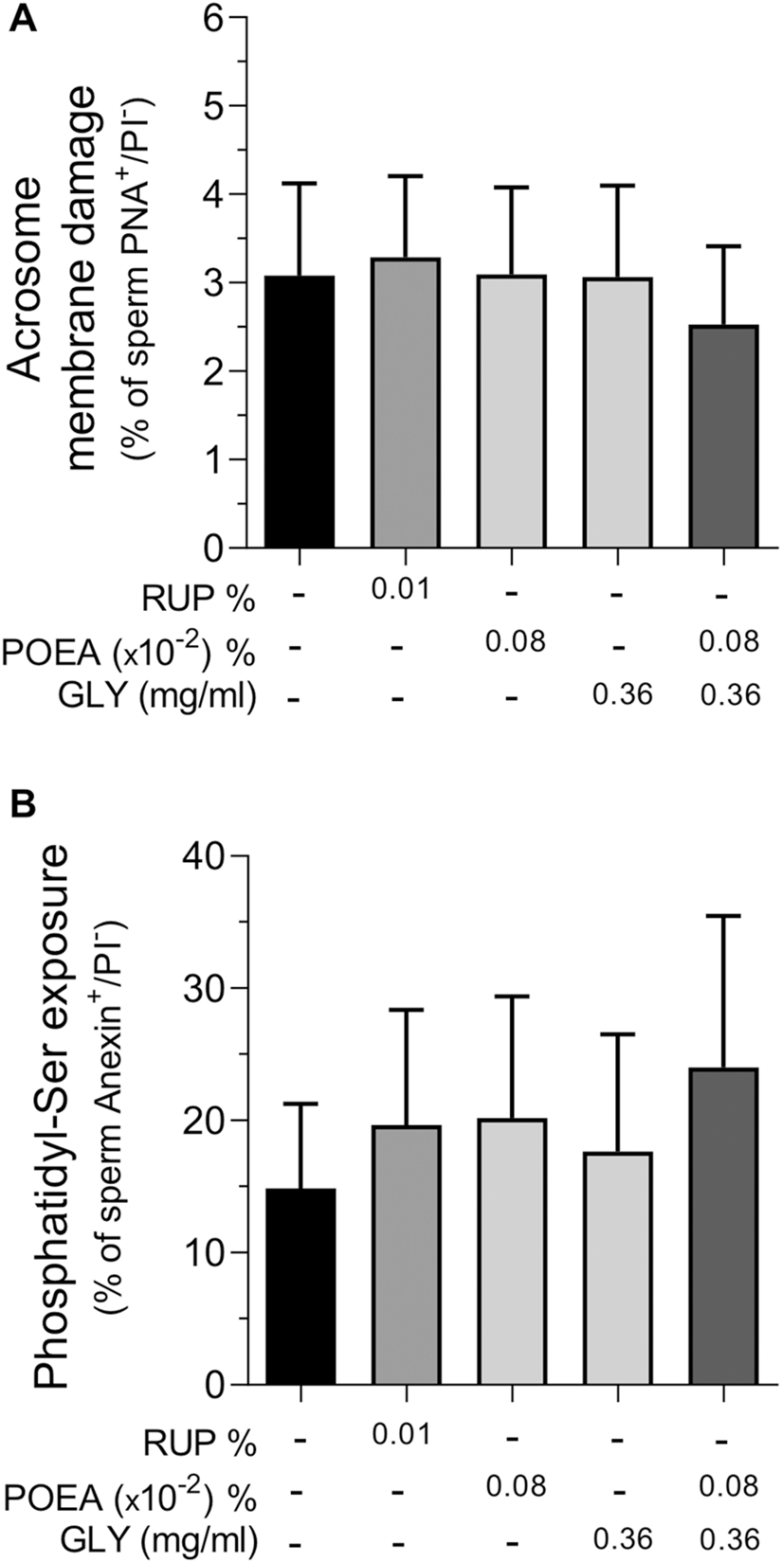
Effects of Roundup Ultra Plus (RUP), glyphosate (GLY) and surfactant polyoxyethylene tallow amine (POEA) on acrosome membrane damage (**A**) and phosphatidyl-serine exposure in the plasma membrane (**B**) in human spermatozoa. Spermatozoa were incubated in BWW for 1 h at 37 °C, 5% CO_2_, in the absence or presence of indicated concentrations of RUP, POEA, GLY or POEA in combination with GLY. The results were expressed as the mean of the percentage of PNA-FITC-positive and PI-negative spermatozoa (n = 5) (**A**), and of Annexin V- FITC positive and PI-negative spermatozoa (n = 4) (**B**) ± standard error of the mean (SEM). No statistical differences were found.

For more in-depth research about RUP effect in human sperm membranes, we studied whether the translocation of the phospholipid phosphatidylserine to the outer face of plasma membrane or the acrosome membrane integrity could be affected by RUP or its components (Fig. 4). Results in Fig. 4A showed that none of the treatments, RUP, POEA or GLY alone or combined GLY and POEA affected significantly the integrity of the acrosome membrane (Fig. 4A) or the phosphatidylserine translocation to the outer leaf of the sperm plasma membrane (Fig. 4B).

### Effects of Roundup Ultra Plus (RUP) and its components, GLY and surfactant POEA, on human sperm acrosome membrane integrity

**Figure 5:**
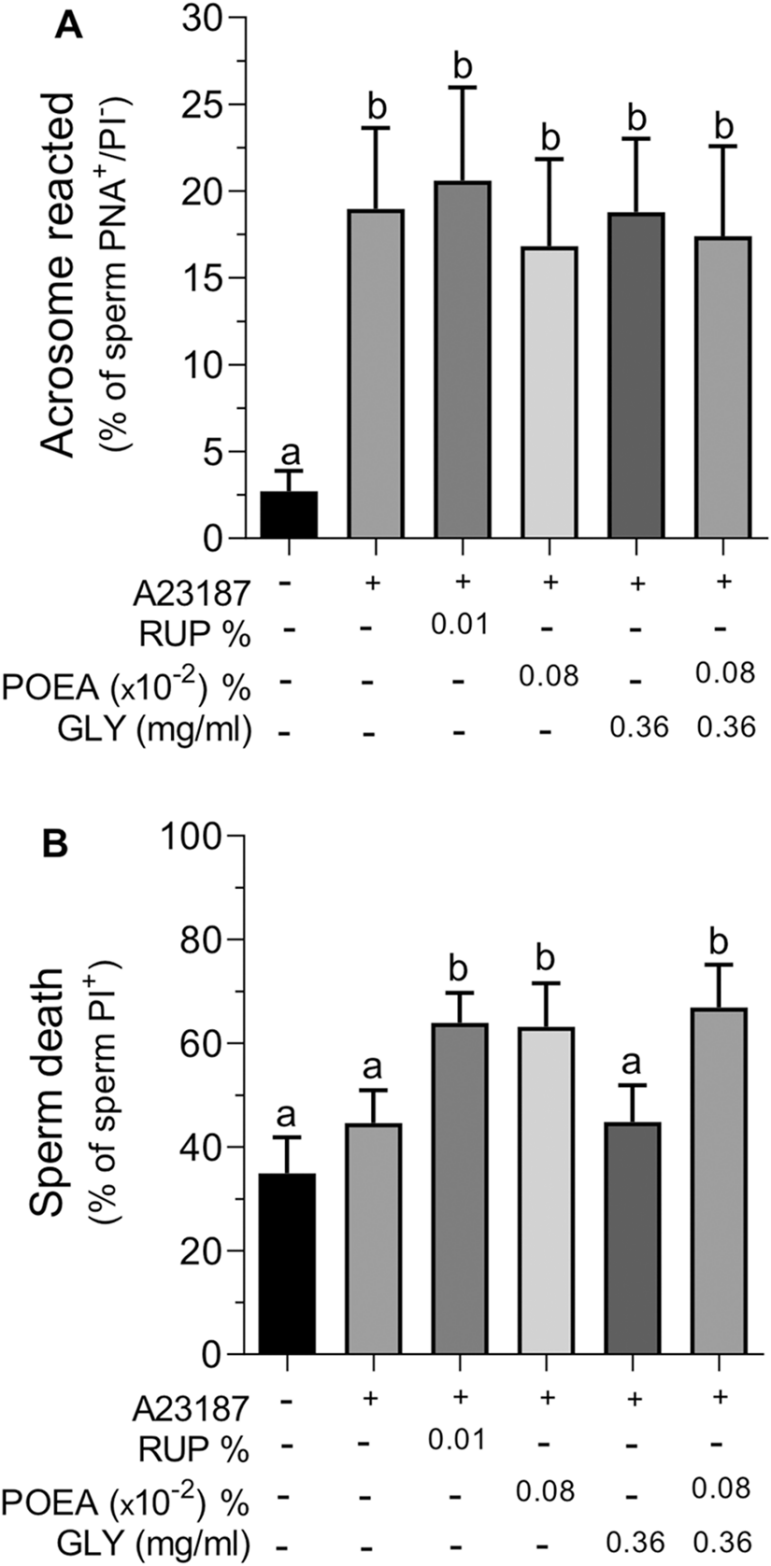
Effects of Roundup Ultra Plus (RUP), glyphosate (GLY) and surfactant polyoxyethylene tallow amine (POEA) on acrosome membrane damaged (**A**) and cell death (**B**) after A23187 treatment in human spermatozoa. Spermatozoa were incubated in BWW for 1 h at 37 °C, 5% CO_2_, in the absence or presence of indicated concentrations of RUP, POEA, GLY or POEA in combination with GLY. This experiment was performed 7 times (n = 7) and the results were expressed as the mean of the percentage of PNA-positive and PI-negative spermatozoa ± standard error of the mean (SEM). Columns with different letters are statistically different from each other, so that for P < 0.05.

An increase in membrane lipid disorganization could be related with sperm capacitation^21^. Therefore, we induced sperm acrosome reaction with calcium ionophore as a way of testing the competence of human spermatozoa to undergo capacitation in the presence of RUP or its components^6^. Results in Fig. 5A showed that incubation with RUP or its ingredients GLY and POEA, alone or combined, did not significantly modify the percentage of acrosome-reacted human spermatozoa (Fig. 5A). However, under these experimental conditions, the herbicide RUP statistically increased the percentage of dead spermatozoa (Fig. 5B), whereas its active ingredient, GLY, did not affect the number of death spermatozoa. However, similar to the RUP effect, POEA treatment also significantly increased the percentage of death spermatozoa that achieved capacitation and subsequent induced-acrosome damaged (Fig. 5B).

### Effects of RUP, GLY and POEA on human sperm mitochondrial activity

**Figure 6:**
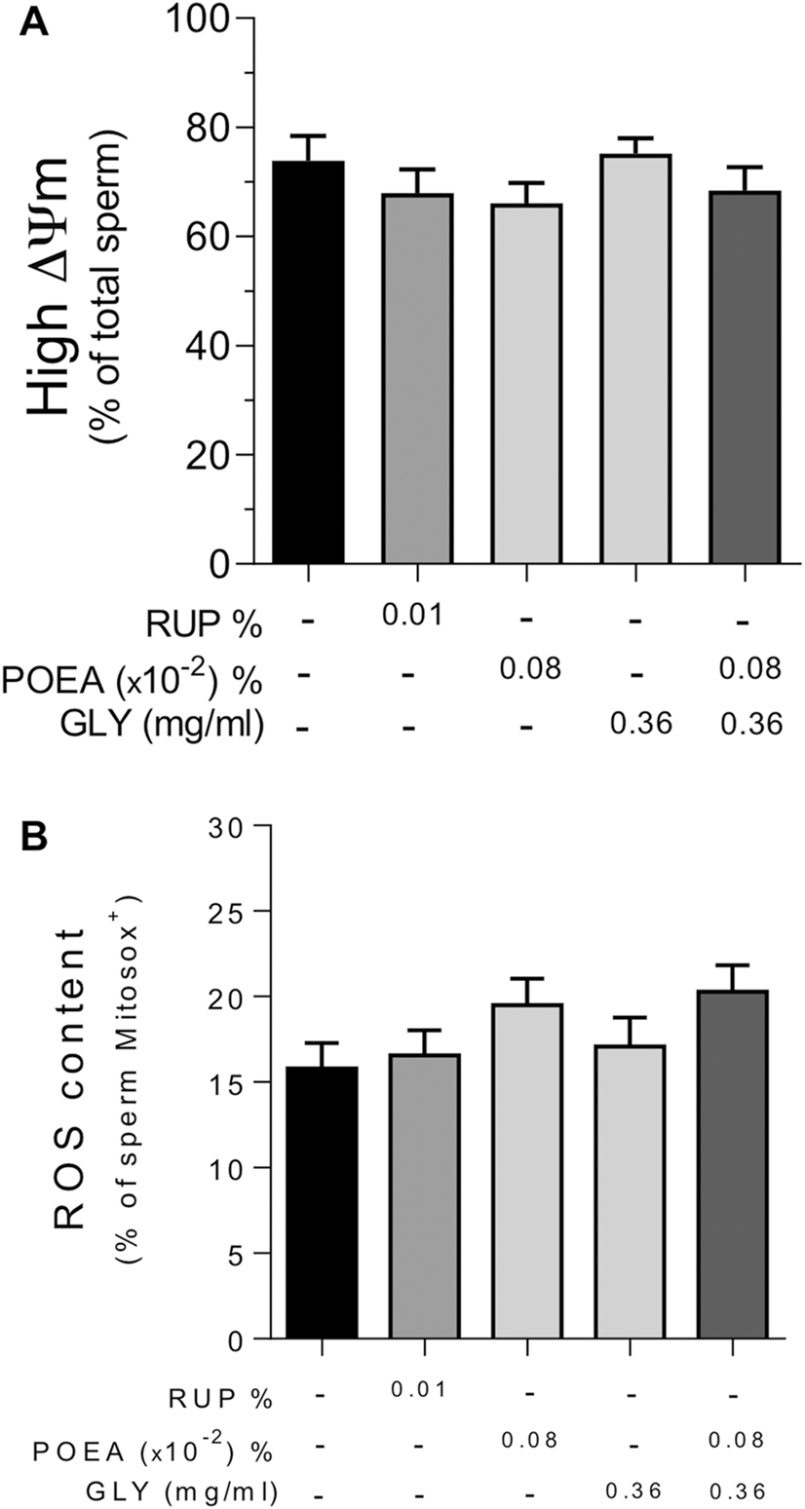
Effects of Roundup Ultra Plus (RUP), Glyphosate (GLY) and surfactant polyoxyethylene tallow amine (POEA) in mitochondrial membrane potential (ΔΨm) (**A**) and high ROS content (**B**) in human spermatozoa. Spermatozoa were incubated for 1 h in BWW at 37 °C, 5% CO_2_, in the absence or presence of different concentrations of RUP, POEA and GLY. Results were expressed as the mean of the percentage of spermatozoa exhibiting higher ΔΨm from the total sperm cells analyzed (**A**), and of the percentage of Mitosox-positive spermatozoa (**B**) ± standard error of the mean (SEM). Each experiment was performed 9 times (n = 9). No statistical differences were found.

Due to the well-known sensitivity of the sperm plasma membrane to oxidative damage and the fact that mitochondria are the main source of ROS in spermatozoa, we studied mitochondrial membrane potential and mitochondrial ROS production. As seen in Fig. 6, neither the treatment with RUP nor its ingredients significantly affected human sperm ΔΨm (Fig. 6A) or the percentage of reactive oxygen species (Fig. 6B).

### Effects of RUP, GLY and POEA on human sperm motility

**Figure 7:**
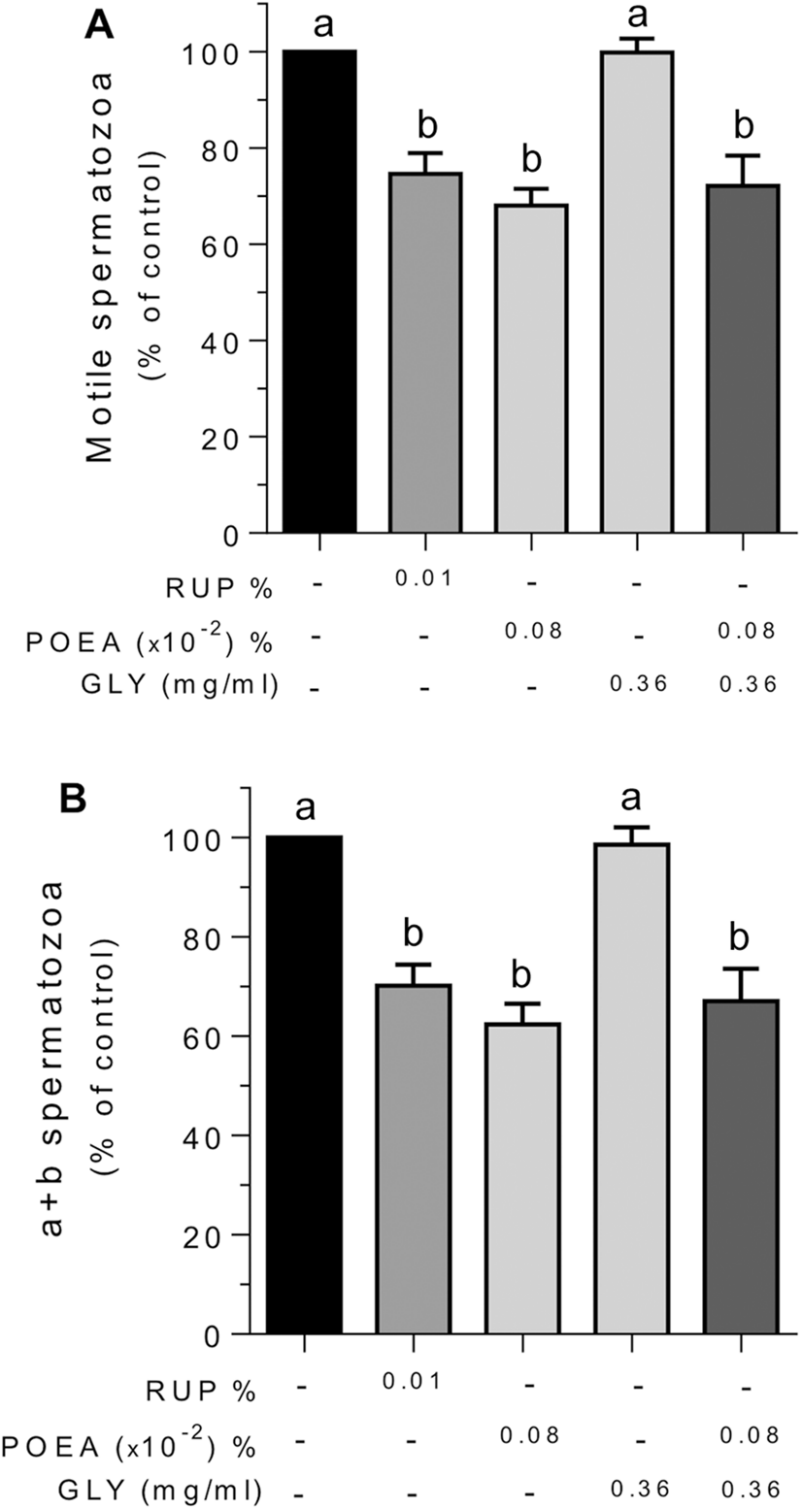
Effects of Roundup Ultra Plus (RUP), glyphosate (GLY) and surfactant polyoxyethylene amine (POEA) in the percentages of motile (**A**) and type a + b human spermatozoa (**B**). Human spermatozoa were incubated 1 h in BWW at 37 °C, 5% CO_2_, in the absence or the presence of indicated concentrations of RUP, GLY and POEA. Each experiment was performed 13 times (n = 13) and results were normalized and expressed as percentage of control in both cases ± standard error of the mean (SEM). Columns with different letters are statistically different from each other, so that for P < 0.05.

We next investigated the impact of the herbicide RUP and its components, alone or combined, on human sperm motility. The treatment with the active ingredient, GLY 0.36 mg mL^−1^, did not modify the percentage of motile (Fig. 7A) and rapid progressive spermatozoa (type a + b according to WHO; Fig. 7B), being the values obtained in each motility parameter similar to the untreated spermatozoa. Interestingly, treatment with RUP 0.01% caused statistically significant decreases in the percentage of motile (Fig. 7A) and rapid progressive spermatozoa (Fig. 7B). In the same way to RUP action, the surfactant POEA 0.0008% caused a similar statistically significant reducing effect in both sperm motility parameters (Fig. 7). Finally, the combination of GLY 0.36 mg mL^−1^ and POEA 0.0008% led to a statistically significant decrease in both the percentage of motile sperm and the percentage of rapid progressive sperm, in a similar way to the effect observed with POEA treatment alone (Fig. 7).

In addition, we evaluated the effects on other motility parameters such as sperm velocities and sperm motility coefficients (Supplementary Table S1).

**Figure 8:**
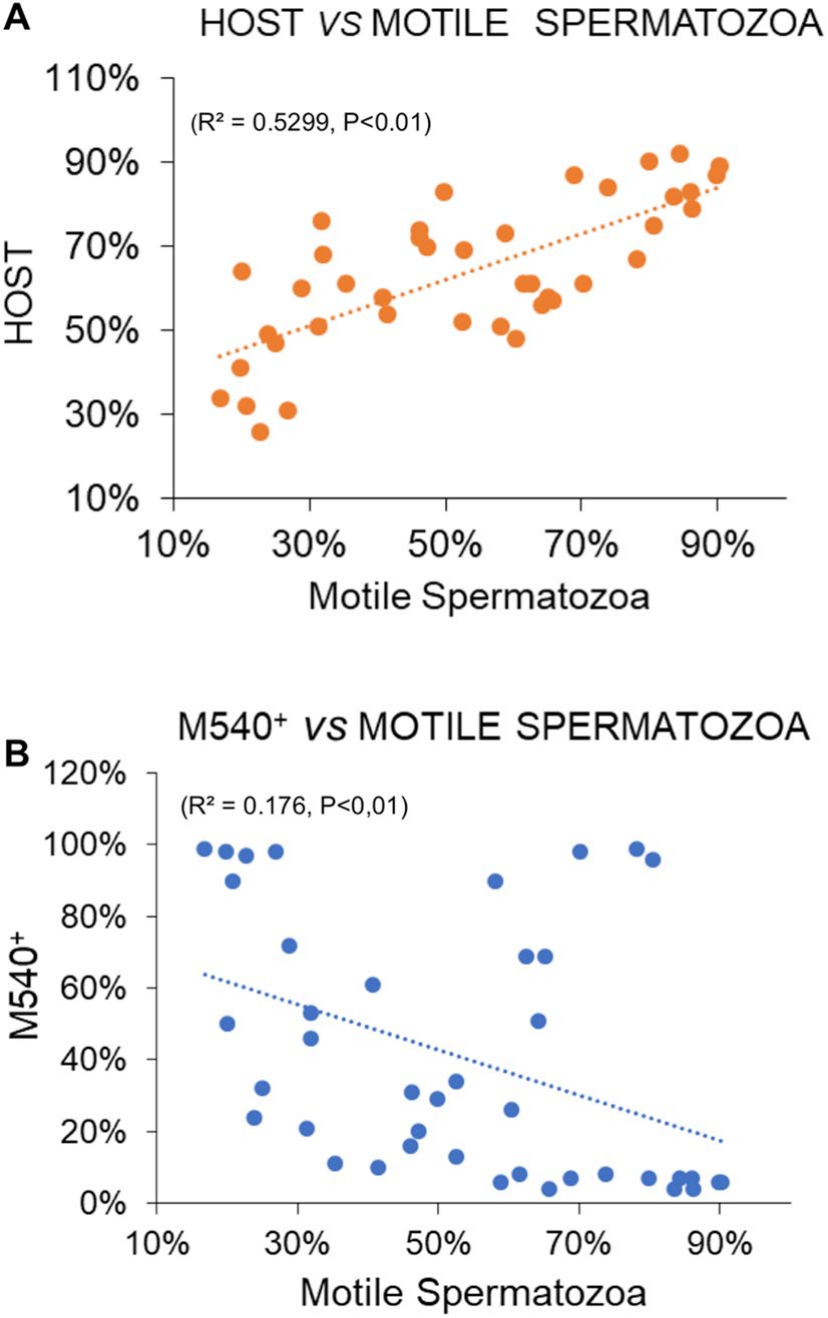
Correlation between human sperm motility and plasma membrane integrity and organization. (**A**) Positive correlation between the functional integrity of sperm plasma membrane evaluated by HOS test and the percentage of motile spermatozoa. (**B**) Negative correlation between the lipid disorganization (M540^+^ spermatozoa) and the percentage of motile spermatozoa. Human spermatozoa were incubated 1 h in BWW at 37 °C, 5% CO_2_, in the absence or the presence of RUP, GLY, or POEA, as described in Material and Methods. Values were result of 7 independent experiments (n = 41) in each correlation. The resultants Pearson coefficients are statistically significant for the positive correlation (**A**) and the negative correlation (**B**), r = 0.728 and − 0.410 respectively (P < 0.05).

We next studied the statistical correlation between sperm motility and sperm membrane fluidity and also between sperm motility and the functional integrity of the plasma membrane analyzed by the HOS test. Figure 8 shows a positive and statistically significant correlation (r = 0.73, *P* < 0.05) between human sperm motility and the percentage of spermatozoa with coiled tails in response to osmotic stress (Fig. 8A). On the other hand, it was found a clear and significant level of negative correlation (r = − 0.41, *P* < 0.05) between the disorganization of plasma membrane lipids and sperm motility (Fig. 8B).

**Figure 9:**
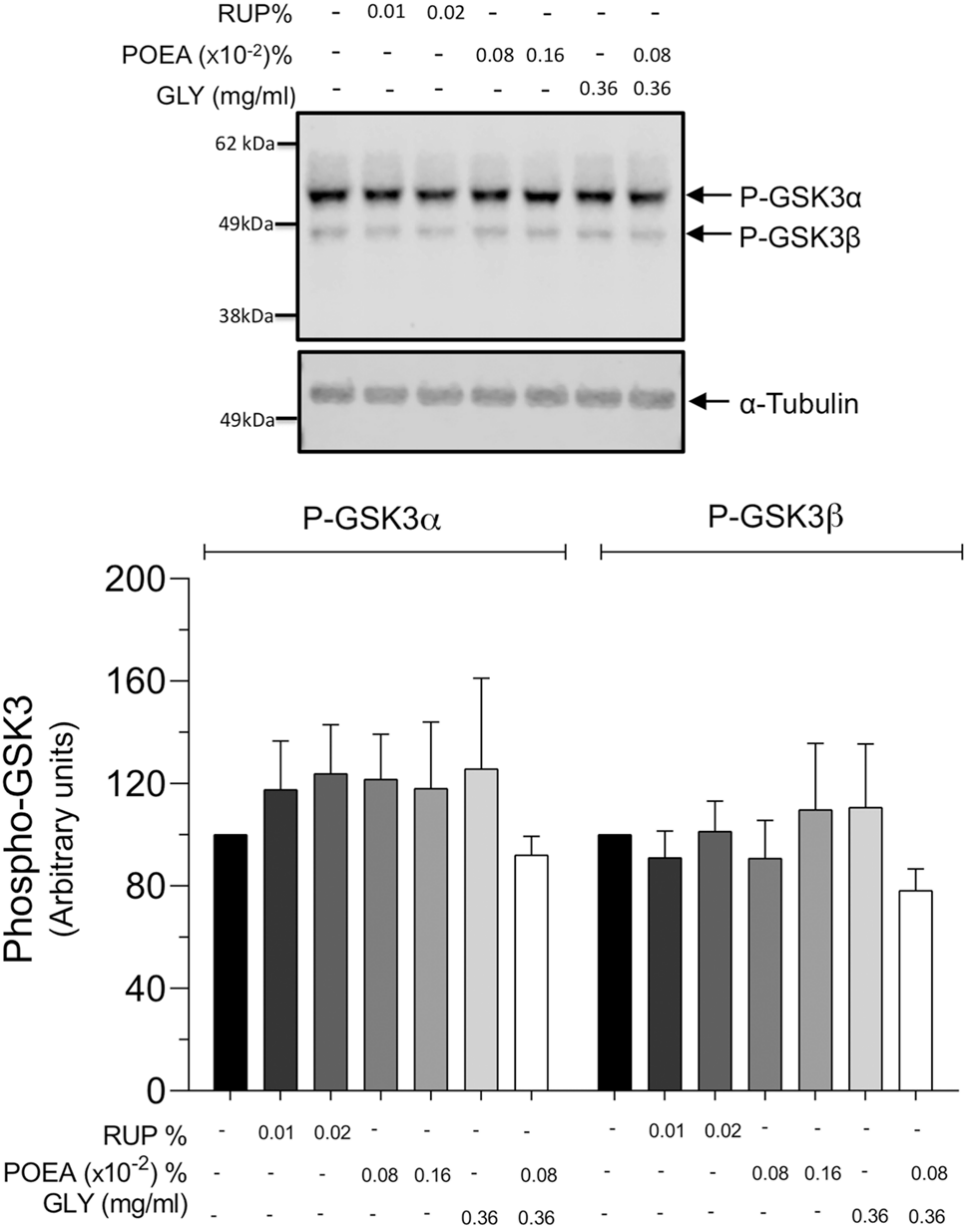
Effects of Roundup Ultra Plus (RUP), glyphosate (GLY) and surfactant polyoxyethylene amine (POEA) in the phosphorylation of GSK3 on human spermatozoa. Spermatozoa were incubated in BWW for 1 h at 37 °C, 5% CO_2_, in the absence or the presence of the herbicide RUP (0.01%, 0.02%) and the equivalent concentrations of its ingredients POEA (0.0008%, 0.0016%), GLY (0.36 mg mL^−1^) or POEA (0.0008%) in combination with GLY (0.36 mg mL^−1^). *Upper panel*: Human sperm proteins (10 μg) were analyzed by western blotting using anti-phospho-GSK3α/β as primary antibody. Arrows indicate the reactive sperm bands corresponding to phosphorylated forms of GSK3α and GSK3β. Each experiment was performed 6 times (n = 6) and representative films are shown. Loading controls using α-Tubulin antibody (lower film) were performed for each experiment. *Lower panel*: Densitometry analysis of Phospho-GSK3α and Phospho-GSK3β bands are shown, and values are expressed as the mean ± SEM of arbitrary units. No statistical differences were found.

Finally, we investigated whether the herbicide RUP, its main components and their combination could have any impact on intracellular signaling pathways relevant to sperm motility such as GSK3α/β. As seen in Fig. 9, the phosphorylation status of GSK3α/β (i.e., its kinase activity levels) in human spermatozoa was not modified by the herbicide RUP or its ingredients.

## Discussion

Scarce and controversial literature exists about the functional impact of the worldwide used glyphosate-based herbicides in human reproductive cells, compared to that existing in other mammal and fish gametes.

To date, the few existing studies in human spermatozoa have focused on herbicide and glyphosate effects, whereas, to our knowledge, there are no studies about the plausible impact of non-active ingredients included in the commercial formulation of the herbicide Roundup. For this reason, this work is pioneer in studying the cellular impact of exposing human spermatozoa to commercial Roundup Ultra Plus herbicide and to its non-active ingredient the surfactant POEA at equivalent concentrations, besides to the combined effect of POEA and glyphosate exposure.

This study demonstrates a clear adverse effect of both, herbicide Roundup Ultra Plus and its surfactant POEA, but no glyphosate, at the human sperm plasma membrane. Supporting this observation, it has been recently found that Roundup adverse effects in other mammalian spermatozoa, as pig^14^ and stallion^17^, primary affect the sperm membrane. Thus, RUP at concentrations that clearly affected sperm function in other mammalian spermatozoa such as pig^14^, increases the lipid membrane disorganization on human spermatozoa. Additionally, the herbicide also reduces its functional integrity evaluated by the HOS test, which determines the ability of sperm membrane to maintain equilibrium between the sperm cell and its environment. Interestingly, these RUP effects at the human sperm plasma membrane are mimicked by POEA at concentrations that are equivalent to RUP. Surprisingly, the exposure to GLY, even at a concentration equivalent to the highest RUP concentration used, does not alter lipid organization or the functional integrity of the plasma membrane. This lack of effect of GLY in human sperm membrane agrees with previous results observed in other mammalian spermatozoa, such as pig^14^ and stallion^17^. However, treatment of human spermatozoa with a combination of GLY and POEA causes the same effects to those obtained with POEA alone or with RUP. These findings clearly suggest that the glyphosate-based herbicide RUP causes an adverse impact at the plasma membrane lipid organization and functional integrity in human spermatozoa, likely due to its surfactant component POEA, the most used surfactant in GLY-based herbicides. Thus, like in stallion spermatozoa^17^, the contribution of RUP active component, glyphosate, at the sperm plasma membrane level is clearly discarded in human spermatozoa. There are no previous data in literature about the impact of RUP or its individual ingredients in human sperm plasma membrane, as preceding works in human sperm were focused only on sperm motility, mitochondrial activity, or DNA fragmentation^15,16^. However, our results agree with previous findings in spermatozoa from other mammal species, as stallion^17^ and pig^14^. In fact, in this later species it has been recently demonstrated that RUP and POEA, at equivalent concentration, but not GLY, clearly alter sperm plasma membrane increasing its lipid disorganization^14,17^. Pointing a membrane impact of POEA in other human cells, it has been demonstrated that this surfactant induces damage in cell membranes leading to permeability changes in human umbilical, embryonic and placental cells^10^, and also disturbs the membrane integrity in other human cell lines^7^. In other species, it has been also reported that POEA induces membrane damages in several mouse cell types causing a high cytotoxicity^22^.

It is interesting to note that the loss of functional integrity of the human sperm plasma membrane caused by RUP and its surfactant does not appear to be complete, as the enzymatic activity of membrane scramblases, measured by outer exposure of phosphatidylserine, is unaffected. Curiously, the adverse effect of RUP and POEA in human sperm membranes seems to be specific of the plasma membrane, as the acrosome membrane remains intact. In contrast with this, RUP, but no GLY, increased acrosome damage in stallion spermatozoa^17^, whereas in pig both, RUP and GLY decreased acrosome membrane integrity in a concentration and time-dependent manner^4^. The explanation, beside the distinct species studied, could be attributed to different incubation times and concentrations of either RUP or GLY.

The impairment of lipid organization and integrity of human sperm plasma membrane by RUP is not due to a cytotoxic impact or to any potential side effect triggered by the herbicide or its surfactant that might compromise human sperm vitality, which remains unaffected by RUP 0.01% or equivalent concentration of POEA. However, a RUP concentration-dependent effect in human sperm viability was found as 2 times higher RUP concentration (0.02%) slightly reduces sperm viability, and 10 times higher RUP concentration (0.1%) causes total sperm mortality (data not shown). Interestingly, the GLY concentration (0.36 mg mL^−1^) that is equivalent to 0.1% RUP had no effect at all in sperm viability, suggesting that GLY is not the RUP ingredient responsible for causing human sperm death. Previous works about RUP in human spermatozoa did not study sperm viability^15,16^ and the most recent only investigated GLY effect, but not RUP^19^. However, sperm DNA fragmentation was unaffected by the herbicide or GLY^16^, suggesting that at the time and concentrations used, RUP or GLY do not exert wide toxic effects in human spermatozoa that might lead to a loss in cell viability. Moreover, the lack of RUP 0.01%, GLY or POEA effects in human sperm viability agrees with previous reports in pig spermatozoa using same concentrations^14^ or with RUP or GLY concentrations below 50 μg mL^−1^, as higher concentrations lead to a dose-dependent decrease in pig sperm viability^4^. These findings together with the fact that RUP, at concentrations similar to this study, caused a decrease in stallion sperm viability^17^, reinforce a previous idea that the glyphosate-based herbicides detrimental effects in mammalian spermatozoa viability are specie and concentration-dependent^4,14,17^.

This work shows that neither RUP, GLY nor POEA modify the mitochondrial membrane potential in human spermatozoa. These data contrast with those reported in human spermatozoa by Anifandis et al.^15^, showing that RUP causes mitochondrial dysfunction and those by Ferramosca et al.^19^, reporting that GLY (0.1–1 μM) reduced the mitochondrial respiration efficiency^19^. These discrepancies can be explained by the fact that Anifandis et al.^15^, used a different RUP concentration (1 mg/L) and also because they evaluated functionally active mitochondria with a different dye, Mitotracker Red CMXROS that stains mitochondria in live cells and its accumulation is dependent upon membrane potential. In this work, we used JC-1 probe that aggregates in mitochondria with high membrane potential. In addition, the lack of effect of GLY in the mitochondrial membrane potential that we have observed contrasts with the work of Ferramosca et al.^19^. An explanation could be the different mitochondrial parameters that were measured in each work and by different techniques (JC-1 versus Clark-type oxygen probe). Moreover, it is difficult to further compare both human sperm studies as Ferramosca et al.^19^, did not study RUP or POEA effect in their experimental conditions. Regarding the effect of RUP in sperm mitochondrial membrane potential from other species, as pig, it has been reported only slight effects by Nerozzi et al.^4^, and by Torres-Badia et al.^14^, whereas in stallion spermatozoa RUP reduced it^17^, suggesting that RUP sensitivity is specie-specific.

In the same way, other human sperm functional processes as ROS generation or acrosome membrane damage, as well as sperm signaling cascades leading to GSK3α/β phosphorylation are unaffected by the treatment with herbicide or its components. There are no existing data in literature about the impact of RUP or its individual ingredients in these human sperm functional parameters. However, in stallion spermatozoa it has been recently reported that RUP, but no GLY, reduced the percentage of intact mitochondria not producing ROS^19^. Interestingly, our results regarding intracellular signaling pathway leading to GSK3α/β phosphorylation in human spermatozoa contrast with those in pig spermatozoa, where herbicide and POEA treatments, but not GLY, inhibited not only GSK3α/β but also PKA substrates phosphorylation^14^. As the concentration of RUP and POEA are the same in both studies, the only explanation plausible can be attributed to specie-specific intracellular effects of the herbicide or surfactant.

Another detrimental action of RUP human spermatozoa observed is a reduction of motility, an effect that is mimicked by its surfactant POEA, in the same way as it has been reported in pig spermatozoa^14^. Interestingly, we have found a clear statistical correlation between the RUP effects at plasma membrane and human sperm motility. Thus, a lower functional integrity of the plasma membrane statistically correlates with lower percentages of motile spermatozoa and higher lipid disorganization statistically correlates with lower sperm motility. This RUP effect in human sperm motility is in agreement with previous works in mammalian spermatozoa reporting that effects of RUP, at lower concentrations than those used as herbicide, lead to an inhibition of sperm motility in pig^4,14^, stallion^19^ and human^15^. Further, our data clearly demonstrate that, in a similar way to the above-mentioned effects in plasma membrane, the impairment of human sperm motility caused by RUP can be attributed to its non-ionic detergent, but not at all to its active ingredient GLY. This finding contrasts with previous results in human spermatozoa^16^ that attribute RUP adverse effect in sperm motility to GLY, although these authors used different RUP and GLY concentrations than those used in this work and, more importantly, they did not study the effect of the surfactant component of RUP in their experimental conditions. Importantly, in our conditions, POEA alone or combined with GLY mimics any human sperm effect caused by RUP. In agreement with this relevant finding, same results, showing that RUP impairment of sperm motility is due to its surfactant component, have been recently described in pig spermatozoa^14^. This discovery is also in line with the study of Nerozzi et al.^4^, in pig and Spinaci et al.^17^, in stallion who pointed out that RUP is more toxic than its main component GLY, suggesting that besides GLY, other RUP non active components should be considered^4,17^.

In conclusion, we believe that future experiments are needed to clarify the intracellular mechanisms by which herbicides as RUP (through their surfactants) cause adverse effects in human spermatozoa plasma membrane and motility, paying careful attention to the surfactant included in its formulation. Surfactants are widely included not only in herbicides, but in different commercial formulations of products that are currently being used in the human daily life (cleaning products). Therefore, the impairment of human sperm function by environmental compounds to which we are daily exposed (surfactants) represents currently an important issue to be addressed in the human male fertility area.

## Methods

### Chemical and sources

Roundup Ultra Plus from Monsanto Europe (Ambers, Belgium); glyphosate potassium salt, M540, PNA-FITC and calcium ionophore A23187 were from Sigma-Aldrich (St Louis, MO, USA); polyethoxylated tallow amine (POEA) from Dr. Ehrenstorfer GmbH (Augsburg, Germany); PureSperm 100 and PureSperm Buffer from Nidacon (Sweden, EU); propidium iodide (PI), SYBR-14, Yo-Pro-1, 5,5′,6,6′–tetrachloro-1,1′,3,3′ tetraethylbenzymidazolyl carbocyanine (JC-1), annexin-V–FITC, MitoSOX Red probe from Thermo Fisher Scientific (Eugene,OR, USA); DC Protein Assays and 2X Laemmli Sample Buffer from Bio-Rad (Hercules, CA, USA); Intercept (TBS) blocking buffer, IRDye 800RD and 680RD secondary antibodies from LI-COR Biotechnology (Bonsai Lab, Alcobendas, Spain). Furthermore, the anti-phospho (Ser 21/9) GSK3α/β (#9331) polyclonal antibody was from Cell Signaling Technology, Inc. (Beverly, MA, USA); the anti-α-tubulin monoclonal antibody (TU-02, #SC-8035) was from Santa Cruz Biotechnology (Santa Cruz, CA, USA). All reagents used to prepare incubation media were purchased from Sigma-Aldrich (St. Louis, MO, USA).

### Spermatozoa incubation media

Biggers, Whitten and Whittingham (BWW): 90 mM NaCl, 4.8 mM KCl, 1.22 mM MgSO_4_, 1.17 KH_2_PO_4,_ 1.7 mM CaCl_2_, 25 mM NaHCO_3,_ 5.5 mM glucose, 0,27 mM sodium pyruvate, 20 mM sodium lactate, 20 mM HEPES and 3 mg mL^−1^ BSA. All media were prepared on the day of use and adjusted to pH 7.45 with an osmolarity of 290–310 mOsm kg^−1^.

### Human semen samples

Thirteen human ejaculates from ten healthy donors (25–35 years old) were obtained by masturbation into specific sterile containers after 3–4 days of sexual abstinence.

Semen samples were prepared and evaluated in line with the recommendations and current values of the World Health Organization (WHO)^20^. After complete liquefaction (between 10 min and 1 h at 37 °C), samples were processed, and the sperm parameters (volume, sperm concentration and percentage of motility) were evaluated using a computer–assisted semen analyser (CASA system). Sperm samples used in this study satisfied the WHO guidelines criteria for normozoospermia (*i.e*., sperm total number of the whole ejaculate > 39 millions; percentages of progressively motile spermatozoa > 32%, morphologically normal spermatozoa > 4%)^20^. The donors previously declared they did not have any condition that would interfere with semen analysis (urogenital infections or systemic diseases) nor a history of smoking, alcohol abuse, or drug consumption; they did not receive supplements or medications before the study.

### Ethical approval

The study was conducted in accordance with ethical guidelines for human samples research and informed and written consent was obtained from all individuals donors included in the study. The University of Extremadura Ethical Committee approved all protocols.

### Human sperm preparation

Before those experiments intended, sperm were washed in 30–60% PureSperm density gradient medium, used in artificial reproduction techniques for separation and purification of motile human spermatozoa. Briefly, aliquots of semen (1 mL) were layered over the upper layer of the density gradient and centrifuged at room temperature for 20 min at 300*g*. Spermatozoa were collected from the 60% fraction and washed once in BWW and centrifuged at RT for 10 min at 400*g*, then the spermatozoa were suspended at 30 × 10^6^ spermatozoa mL^−1^ in BWW.

Depending on each experimental procedure, 150 or 500 µL of spermatozoa samples were incubated 1 h at 37 °C with 5% of CO_2_ in the absence (control) or presence of Roundup Ultra Plus (RUP) that was previously diluted 1/1000, 1/5000, 1/10,000 and 1/1,000,000 times, yielding final RUP concentrations (v/v) of 0.1%, 0.02%, 0.01% and 0.0001%, respectively. The commercially available Roundup Ultra Plus contains 36% (w/v) of the active ingredient GLY and 6–8% (w/v) of a surfactant. Glyphosate (GLY) concentrations used are equivalent to the highest and lowest RUP dilutions (0.36 mg mL^−1^, 0.36 µg mL^−1^). The three higher above-mentioned dilutions of the commercial RUP yield a final surfactant, polyoxyethylene amine (POEA), concentrations of 0.008%, 0.0016% and 0.0008%, respectively.

These concentrations of the active and non-active ingredients of RUP, which resulted after different RUP dilutions, were so-called equivalent concentrations of RUP and used for the treatment of human spermatozoa.

In order to minimize possible experimental variations, every condition/treatment studied was performed in semen samples obtained from the same human ejaculate.

### Evaluation of spermatozoa motility

After incubation with RUP or its different components, 6 μl of spermatozoa sample were placed in a 37 °C pre-warmed Spermtrack sperm counting chamber with 20 µm depth (Proiser R + D, Paterna, Valencia, Spain).

Spermatozoa images were taken using a microscope equipped with a 10× negative-phase contrast objective, with a heated stage, and a CCD camera that takes 25 consecutive digitalized images obtained during 1 s from at least 4 different fields and the minimum number of spermatozoa evaluated in each semen sample was at least 300. Digitalized images were analyzed using a Computer Assisted Semen Analysis system, specifically the ISAS system (Integrated Semen Analysis System, Proiser R + D, Paterna, Valencia, Spain).

The settings of the CASA system were as follows: frame rate, 60 Hz; frame acquired, 25; rapid spermatozoa with average path velocity > 35 μm s^−1^, progressive spermatozoa with a straightness threshold > 80%.

### Flow cytometry analysis

Flow cytometry analysis was performed using an ACEA NovoCyte flow cytometer (ACEA Biosciences, Inc., San Diego, CA, USA) equipped with a three detection channels for blue laser (488 nm): BL-1 (530 ± 30 nm band pass filter); BL-2 (572 ± 28 nm band pass filter) and BL-4 (675 ± 30 nm band pass filter) and a detection channel for a red laser (640 nm): BL-3 (660 ± 20 nm band pass filter), as previously described^23^. Flow cytometry experiments and data analyses were performed using ACEA Novo Express software (ACEA Biosciences, Inc., San Diego, CA, USA). Fluorescence data were represented in a logarithmic scale.

#### Analysis of spermatozoa viability by flow cytometry

Fluorescent staining using SYBR-14 and propidium iodide (PI) was performed to measure sperm viability. Briefly, 2.5 μl of SYBR-14 (2 μM) and 5 μl of PI (480 μM) were added to 10 μl of spermatozoa (30 × 10^6^ cells mL^−1^) diluted with 240 μl of BWW, until a final concentration of 20 nM for SYBR-14 and 9.6 μM for PI. Then, the samples were incubated for 20 min at room temperature (RT) in darkness and analyzed in the flow cytometer. After excitation at 488 nm, the fluorescence values of SYBR-14 were collected in the laser-excited fluorescence channel (BL1) and PI fluorescence was collected in the BL3 channel. Results of viable spermatozoa were expressed as the average of the percentage of SYBR-14 + and PI − spermatozoa ± standard error of the mean (SEM).

#### Analysis of sperm mitochondrial membrane potential (ΔΨm) by flow cytometry

Mitochondrial membrane potential variations (ΔΨm) were evaluated using the specific probe JC-1 (5,5′,6,6′–tetrachloro-1,1′,3,3′ tetraethylbenzymidazolyl carbocyanine iodine). This lipophilic cationic fluorochrome was present as protomeric aggregates in mitochondria with high membrane potential that emit in orange (585), whereas in mitochondria with low membrane potential, JC-1 was present as monomers that emit in green (525 nm), when is excited at 488 nm.

The experimental procedure consisted of diluting 10 μl of spermatozoa (30 × 10^6^ cells mL^−1^) in 240 μl of BWW containing 1.5 μM of JC-1, mixed an incubated at 37 °C for 1 h. The samples were mixed again before flow cytometry analysis. The fluorescence values of monomer probe JC-1 were collected in the channel BL1 and JC-1 protomeric aggregates in the channel BL2. The percentage of orange-stained cells was recorded and considered the population of spermatozoa with a high mitochondrial membrane potential. Results were expressed as the average percentage of orange-stained (high ΔΨm) spermatozoa ± standard error of the mean.

#### Analysis of the degree of sperm plasma membrane lipid organization by flow cytometry

Fluorescent staining using the probes merocyanine M540, as a membrane lipid fluidity marker, and Yo-Pro-1, as a marker of changes in plasma membrane permeability (commonly associated with cell death) was performed. Briefly, 10 μl of spermatozoa (30 × 10^6^ cells mL^−1^) were diluted in 240 μl of BWW containing 75 nM of Yo-Pro-1 and 6 μM of M540 and incubated at 37 °C for 10 min. Then, remixed before flow cytometry analysis. The fluorescence values of probes Yo-Pro-1 and M540 were collected on both BL-1 and BL-2 channels, respectively. Labeled spermatozoa were categorized as (i) viable cells (Yo-Pro-1^−^, M540^+^), and (ii) non-viable cells (Yo-Pro-1^+^). Results were expressed as the average percentage of M540^+^/Yo-Pro-1^−^ spermatozoa ± standard error of the mean.

#### Analysis of human spermatozoa acrosome damage by flow cytometry

The population of spermatozoa with damaged acrosome was assessed after staining these germ cells with fluorescein isothiocyanate-conjugated peanut agglutinin (PNA-FITC) as a specific marker for acrosome membrane status and PI as a marker for cell death. Aliquots of 10 μL of each semen sample (30 × 10^6^ cells mL^−1^) were incubated at RT in darkness for 5 min with 5 μL of PNA-FITC (1.25 μg mL^−1^) and 5 μL of PI (24 μm). Then, 150 μL of BWW was added to each sample and mixed before flow cytometry analysis. The fluorescence value of probe PNA-FITC was collected in the channel BL1 and the fluorescence value of PI was collected in the BL3 channel. Results were expressed as the average of the percentage of PNA + and PI − spermatozoa ± standard error of the mean.

#### Analysis of human sperm phosphatidylserine externalization at the outer leaflet plasma membrane by flow cytometry

The study of phosphatidylserine (PS) externalization in plasma membrane spermatozoa was performed using annexin-V–FITC to specifically detect PS translocation from the inner to the outer leaflet of the human sperm plasma membrane. Briefly, 10 μL (30 × 10^6^ cells mL^−1^) sperm cells were diluted in 90 μL of Annexin binding buffer: 96 mmol L^−1^ NaCl, 4.7 mmol L^−1^ KCl, 0.4 mmol L^−1^ MgSO4, 0.3 mmol L^−1^ NaH2PO4, 5.5 mmol L^−1^ glucose, 1 mmol L^−1^ sodium pyruvate, 21.6 mmol L^−1^ sodium lactate, 20 mmol L^−1^ HEPES (pH 7.45), and 2.5 mmol L^−1^ CaCl_2_, followed by incubation at RT for 15 min with 5 μL of annexin-V-FITC and 2,5 μl of PI 480 μM. Finally, 150 μL of BWW were added to each sample and mixed before flow cytometry analysis. The fluorescence values of probes annexin V-FITC and PI were collected in the BL1 channel and BL3 channel, respectively. The results were expressed as the average of the percentage of annexin-V-FITC + /PI − spermatozoa ± standard error of the mean.

#### Analysis of mitochondrial reactive oxygen species production in human sperm by flow cytometry

MitoSOX Red was used to measure mitochondrial reactive oxygen species (ROS) production. MitoSOX Red reagent enters the mitochondria and is oxidized by the superoxide anion. The resulting oxidation product becomes highly fluorescent upon binding to nucleic acids. Briefly, 10 μL from each sperm sample (30 × 10^6^ cells mL^−1^) were mixed and incubated with 0.5 μL of MitoSOX (2 μM) at 37 °C for 15 min. The samples were mixed again before flow cytometry analysis, and the fluorescence values of MitoSOX were collected in the BL2 channel. Results were expressed as the mean percentage of MitoSOX-positive sperm ± standard error of the mean.

### Evaluation of sperm plasma membrane integrity by the hypo-osmotic swelling test (HOS test)

The HOS test predicts membrane integrity by determining the ability of the sperm membrane to maintain equilibrium between the spermatozoa cell and its environment. Influx of the fluid due to hypo-osmotic stress causes the sperm tail to coil. A higher percentage (≥ 60%) of swollen spermatozoa indicates the presence of spermatozoa having a functional and intact plasma membrane^24^.

Briefly the solutions were prepared using distilled water. Solution A: 1.5 mM fructose and Solution B: 1.5 mM sodium citrate. Then these solutions were mixed (equal volume) and stored in 1 mL aliquots at − 20 °C until use. Before use, the HOS solutions were incubated at 37 °C for 10 min then 0.1 mL of semen samples were added, mixture and incubated for at least 30 min but not longer than 1 h. After incubation, the tubes were mixed gently and 6 μl of spermatozoa sample were placed in a Makler counting chamber and observed and counted on microscope under phase contrast, at least a total of 200 spermatozoa per sample, including both swollen and non-swollen spermatozoa. Results were expressed as the mean percentage of swollen spermatozoa ± standard error of the mean.

### Analysis of human spermatozoa phosphorylated proteins by western blotting

Spermatozoa (500 µL) were centrifuged at 5,000 g for 1 min at RT and washed in PBS medium and centrifuged again. Pellet was suspended in 30 μl of Laemmli Sample Buffer (2 ×), incubated for 10 min in constant rotation at 4 °C and then centrifuged at 10,000*g* for 10 min at 4 °C. The protein concentration of the supernatant was determined using a Bio-Rad DC Protein Assay. After protein concentration analysis, 2-mercaptoethanol was added to the sperm lysates until final concentration of 3% v/v before heating for 5 min at 95 °C and stored at − 20 °C.

Sperm proteins (10 μg) were resolved using 10% SDS-PAGE. Electrophoresis was run at 90 V during the first 20 min, then at 145 V for another 90 min at room temperature in 1 × running buffer. After electrophoresis, proteins were transferred to nitrocellulose membranes at 380 mA for 2.5 h, then were blocked for 1 h using Intercept (TBS) blocking buffer containing 0.2% Tween-20. Membranes were then incubated at 4 °C overnight using anti-phospho-GSK3α/β (1:1.000) or anti-α-tubulin (1:5000) antibodies. Later, membranes were washed and incubated with the appropriate secondary antibody IRDye 800RD or 680RD as indicated by de manufacturer. Fluorescence was detected using an Odyssey Fc Imaging System (LI-COR Biotechnology), and all bands were quantified using the Image Studio™ software from LI-COR.

### Statistical analysis

In order to show if the differences between the different treatments and dilutions were statistically significant, hypothesis tests were carried out. Data were analyzed for normal distribution with a Kolmogorov–Smirnov test and for homoscedasticity with a Levene test. Differences were determined by a parametric test, as one-way analysis of variance (ANOVA) followed by post-hoc Tukey. Two-sided bivariate Pearson correlation was used to study the correlation between the lipid disorganization (M540-positive and Yo-Pro-1-negative spermatozoa) and the functional integrity (HOS test) of the human sperm plasma membrane and between both parameters and the percentage of motile spermatozoa. All data was shown as the mean ± standard error of the mean (SEM). All analyses were performed using SPSS v27 for Windows software (SPSS Inc. Chicago, IL). Statistical significances were set at p values lower than 0.05.

## Supplementary Information

Supplementary Table 1.

Supplementary Figure 1.

Supplementary Information 3.

Supplementary Information 4.

## Data Availability

The datasets used and/or analysed during the current study available from the corresponding author on reasonable request.
