# Supplementary material for: The adverse impact of herbicide Roundup Ultra Plus in human spermatozoa plasma membrane is caused by its surfactant
Source: Sci Rep. 2022 Jul 29;12:13082. doi: 10.1038/s41598-022-17023-3 (PMC9338072; doi:10.1038/s41598-022-17023-3)
Supplement: Supplementary file 1 — Supplementary Table 1. [file 41598_2022_17023_MOESM1_ESM.pdf]

The adverse impact of herbicide Roundup® Ultra Plus in human spermatozoa plasma membrane is caused by its surfactant

Mercedes Torres-Badia, Soraya Solar-Malaga, Rebeca Serrano, Luis J Garcia-Marin, M. Julia Bragado

Supplementary Table 1. Effects of Roundup® Ultra Plus (RUP), glyphosate (GLY) and surfactant polyoxyethylene amine (POEA) in human sperm motility descriptor parameters.

Treatment	Motile spermatozoa (%)	Progressive spermatozoa (%)	VCL (%)	VSL (%)	VAP (%)	LIN %	STR %	WOB%	BCF	ALH
**Control**	63.85±5.64	56.77±5.32	87.65±3.83	53.38±3.14	58.65±3.09	60.35±1.51	84.56±0.80	67.73±1.32	9.26±0.24	2.92±0.09
**RUP 0.01%**	49.88±6.34	41.90±5.74	83.16±3.95	47.83±4.06	53.03±3.67	56.25±2.88	82.22±1.84	64.33±2.21	8.70±036	2.92±0.14
**POEA 0.0008%**	45.48±5.74	37.34±5.24	74.77±3.20	44.05±2.87	48.25±2.62	57.05±2.30	83.36±1.81	64.65±1.60	8.81±0.34	2.69±0.11
**Glyphosate 0.36mg/mL**	64.27±6.25	56.39±5.92	88.34±3.57	54.16±2.81	59.17±3.03	60.21±1.32	84.30±0.80	67.24±1.14	9.42±0.20	2.90±0.09
**Glyphosate 0.36mg/mL + POEA 0.0008%**	49.04±7.10	40.64±6.24	73.75±3.86	43.31±3.50	46.99±3.33	56.93±2.93	84.20±1.54	63.95±2.39	8.52±0.39	2.68±0.13

Human spermatozoa were incubated in BWW 1 h at 37 °C, 5% CO_2, in the absence or presence of indicated concentrations of RUP, GLY, POEA or POEA plus GLY. Sperm kinematic parameters were evaluated by ISAS® system: curvilinear velocity (VCL, μm/s), linear velocity (VSL, μm/s) mean velocity (VAP, μm/s), linearity coefficient (LIN, %), straightness coefficient (STR, %), wobble movement coefficient (WOB, %), mean lateral head displacement (ALH, μm) and frequency of head displacement (BCF, Hz). This experiment was performed 13 times (n = 13) and values are expressed as the mean ± standard error of the mean (SEM).
