# Supplementary material for: The adverse impact of herbicide Roundup Ultra Plus in human spermatozoa plasma membrane is caused by its surfactant
Source: Sci Rep. 2022 Jul 29;12:13082. doi: 10.1038/s41598-022-17023-3 (PMC9338072; doi:10.1038/s41598-022-17023-3)

The adverse impact of herbicide Roundup® Ultra Plus in human spermatozoa plasma membrane is caused by its surfactant

Mercedes Torres-Badia, Soraya Solar-Malaga, Rebeca Serrano, Luis J Garcia-Marin, M. Julia Bragado*.

Figure S1. Study design. From the preparations of the human sperm samples and their treatments, to the different parameters analyzed. BWW: Biggers, Whitten and Whittingham; RUP: Roundup® Ultra Plus; POEA: polyoxyethylene amine; GLY: potassium glyphosate; CASA: Computer Assisted Semen Analysis; HOST: hypo-osmotic swelling test.

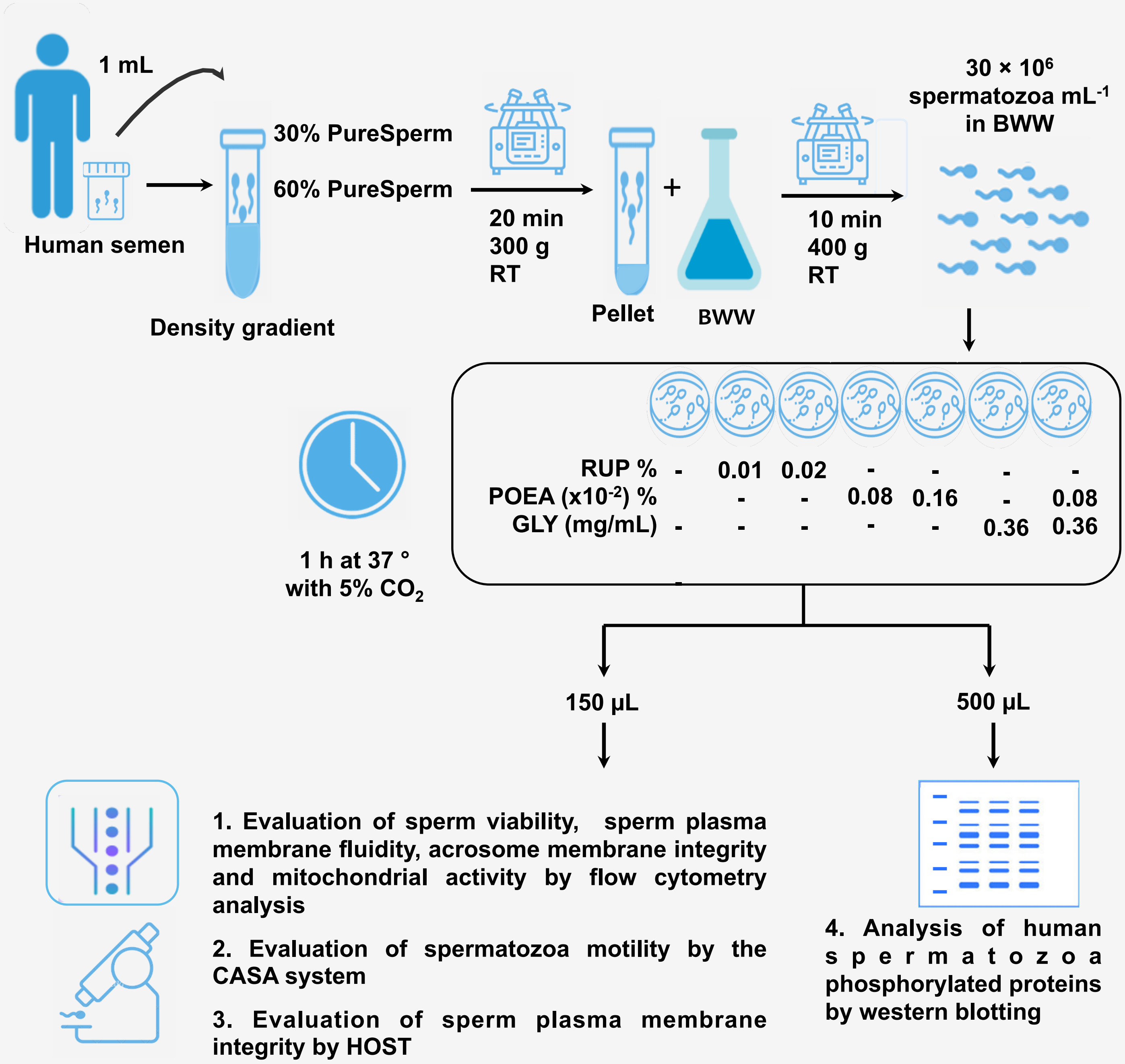

Supplement: Supplementary file 2 — Supplementary Figure 1. [file 41598_2022_17023_MOESM2_ESM.pdf]
